# Basal and Infectious Enteritis in Broilers Under the I See Inside Methodology: A Chronological Evaluation

**DOI:** 10.3389/fvets.2019.00512

**Published:** 2020-02-14

**Authors:** Adrien W. D. Sanches, Bruna L. Belote, Paulo Hümmelgen, Ana C. W. Heemann, Igor Soares, Aline Tujimoto-Silva, Amanda G. C. Tirado, Anderson F. Cunha, Elizabeth Santin

**Affiliations:** ^1^Laboratório de Microbiologia e Ornitopatologia, Universidade Federal do Paraná, Curitiba, Brazil; ^2^Laboratório de Bioquímica e Genética Aplicada, Departamento de Genética e Evolução, Centro de Ciências Biológicas e da Saúde, Universidade Federal de São Carlos, São Carlos, Brazil

**Keywords:** microscopic enteritis, basal enteritis, ISI, regenerative inflammation, inflammatory chronology

## Abstract

Recently, the inflammation of the intestinal mucosa has been related to many diseases in humans and animals. The concept of Microscopic Enteritis (ME) used in human pathology through the Marsh classification system has no counter-part in veterinary medicine. In poultry science, the I See Inside (ISI) methodology, unlike the current linear measures of villi and crypts, generates possibilities to describe and understand the avian ME. Through specific parameters, graded from 0 to 3, the model links proliferative and/or inflammatory reactions in the intestinal layers to some loss in performance. Herein, two trials were conducted in order to describe the development of ME through the ISI methodology in chickens challenged or not with *Eimeria* spp. and *Clostridium perfringens*. In each trial, a total of 64 birds were divided in 2 treatments with 4 replicates containing 8 birds each: non-challenged (NCH) and challenged (CH) through gavage with an *Eimeria* spp. vaccine at 1 day of age and 10^8^ CFU/mL of *Clostridium perfringens* administered at 10, 11, and 12 days of age. At 7, 14, 21, and 28 days of age birds were euthanized and samples of ileum and liver were collected for ISI evaluation, cytokines and presence of macrophages, CD4+ and CD8+ cell. The results allowed the description of the avian Microscopic Enteritis and of its two basic components: a basal enteritis (BE) in NCH broilers, over which the infectious enteritis is developed in CH birds. In addition, the chronology of ME translated by the ISI methodology parameters were associated to losses in zootechnical performance.

## Introduction

The concept of Microscopic Enteritis (ME) has been applied in human pathology to describe an inflammatory process that occurs without macroscopic manifestations. This enteric condition has been described through the Marsh Classification, where scores that range from 0 to 3 describe the intensity of established morphological parameters such as inflammation in lamina propria and epithelial inflammation (1–3). In veterinary medicine, similar concepts are still not currently applied, except for the I See Inside (ISI) methodology, which is a similar model that applies specific parameters to evaluate the intestinal mucosa and correlates histologic changes to some losses in zootechnical performance (4, 5). Unlike in mammals, the strong proliferative reaction of the epithelium specific to the avian mucosa has been described in parameters as the enterocyte and goblet cells proliferation. The ISI methodology concurs with the score intervals proposed in the Marsh classification, describing the microscopic enteritis (ME) in broilers in a more dynamic way than the traditional linear measurements of villi and crypts, still used in the current poultry science. Since it encompasses inflammation in larger scale, it also accepts the concepts of ME up to score 3, after which the gross lesions start to appear. Under experimental conditions, the ME is better described through comparisons among challenged and non-challenged animals, when the real and specific impact of certain challenging element can be verified through differences in histopathological conditions. This study aimed to apply the ISI methodology in the description of a constant and unspecific microscopic basal enteritis (BE) observed in non-challenged broilers raised in a controlled and clean experimental environment, also evaluating how this condition changes with the challenge by *Eimeria* spp. and *Clostridium perfringens*.

## Materials and Methods

The two trials performed in this study were approved by the Institutional Animal Use Ethics Committee of Agricultural Sciences of the Federal University of Parana (Protocols 041/2016 and Protocol 081/2016).

### *In vivo* Experiment: Birds, Experimental Design, Diet, and Housing

The experiments were conducted in previously disinfected isolated rooms with negative pressure, containing vertically stacked cages (replications) with sterilized wood shaving litter (to avoid external contamination), nipple drinkers, and automatic control of temperature and lighting. In both trials, 64 male broilers Cobb® 500 were housed from 1 to 28 days of age, totalizing 128 birds within the two trials. Both experiments followed a randomized design with 2 treatments with 4 replicates containing 8 birds each. The treatments were: non-challenged (NCH) and challenged (CH) with *Eimeria* spp. and *Clostridium perfringens*. Animals were maintained in adequate temperature for their age, with feed and water *ad libitum*. The diet was mashed and based on corn and soymeal, following Brazilian nutritional recommendations for poultry, following Rostagno (6). No fasting was induced before the euthanasia.

### Zootechnical Performance

At 1 day of age, birds were distributed into groups in a way to obtain an equal initial body weight in each cage (replicate) of each group. Birds and feed were weekly weighed (time 0, 7, 14, 21, and 28 days) to evaluate feed intake (FI), body weight gain (BWG), and feed conversion ratio (FCR).

### Challenge and Sampling

On the first day of age, all birds from the CH group received 0.5 ml of a live attenuated *Eimeria* vaccine (Bio-Coccivet®–Biovet) provided without any additional dilution by gavage, guaranteeing that the product was released in the crop. The vaccine contained oocysts of *Eimeria acervulina, Eimeria brunetti, Eimeria maxima, Eimeria necatrix, Eimeria praecox, Eimeria tenella*, and *Eimeria mitis* and the administered dose was 15 times higher than the manufacturer recommendation (7.1 × 10^4^ oocysts per bird). At 10, 11, and 12 days of age, 1 ml of a bacterial suspension containing 10^8^ CFU/mL of *Clostridium perfringens* (CP) was also administered by gavage to the birds of the CH group. The strain was obtained from an outbreak of necrotic enteritis in a brazilian poultry farm. The bacterium was isolated, typified by standard biochemical tests and kept in room temperature in cooked meat medium with periodical replications. The pure culture of the bacterium was transferred to a plate dish with blood agar inside an anaerobic jar for 24 h. One sachet of Oxoid AnaeroGen 2.5L (Thermo Scientific™) was used to induce the anaerobic condition. After the incubation, samples of the grown colonies were inserted in a tube with 9 ml of thioglycolate broth for 5 h at 42°C. Another replication was made in blood agar for 24 h at 42°C. Then, samples were inoculated in tubes with 20 mL of thioglycolate and cultured for 5 h at 42°C to obtain the final inoculum. The counting of 10^8^ CFU/mL considered the percent of colonies tested after the standard dilutions for UFC counting.

At 7, 14, 21, and 28 days of age, 4 birds per treatment (one bird per replicate) were euthanized by cervical dislocation following the brazilian regulation on animal experimentation and samples of ileum were collected and prepared for histological analysis following Belote et al. (5). At 7, 14, and 21 days, samples of ileum and liver were also collected for the evaluation of cytokines mRNA expression. The liver samples were crushed with blade (to increase the efficacy of the preservation) and inserted in sterilized Eppendorf tubes containing 1 ml of RNA*later*™ Stabilization Solution (INVITROGEN, AM7021). The mucosa was scraped with blade from the ileum sections and also inserted in sterilized Eppendorf containing the same mentioned solution for RNA preservation. After the collection, the tubes were rattled to guarantee a proper mix of the content.

### Cytokine Expression

For the evaluation of cytokines mRNA expression, total RNA from ileum and liver was isolated using TRIzol reagent (15596-018, Invitrogen, Carlsbad, CA, USA) following the manufacturer's procedure instructions. Turbo-DNAse kit (AM1907, Applied Biosystems, Foster City, CA, USA) was used for the digestion of contaminant DNA in the collected samples. RNA concentrations were quantified by NanoDrop Spectrophotometer (ND1000, Thermo Scientific, Bonn, Germany). A total of 1 μg of RNA was reverse transcribed with High Capacity cDNA Reverse Transcription kit (Applied Biosystems-Thermo Fisher Scientific, Waltham, Massachusetts, USA). RT-qPCR was conducted using Power Sybr® Green PCR Master Mix (Applied Biosystems-Thermo Fisher Scientific, Waltham, Massachusetts, USA) on StepOne Plus Real Time PCR System (Applied Biosystems-Thermo Fisher Scientific, Waltham, Massachusetts, USA). All the primers were designed using OligoAnalyzer 3.1 (Integrated DNA Technologies, Coralville, Iowa, USA) and are listed in Table 1. The concentration of primers was optimized prior to the efficiency curve reaction with efficiency ranging from 95 to 105%. Relative fold change in mRNA quantity was calculated according to 2^(−ΔΔCt)^ method and all values were normalized to the expression of the avian Glyceraldehyde 3-phosphate dehydrogenase (GAPDH) gene.

**Table 1:** Primers used for cytokines gene expression.

**Cytokine**	**F 5′-3′**	**R 5′-3′**
IL-10	AAGCAGATCAAGGAGACGTTC	GATGAAGATGTCGAACTCCCC
IL-8	CAGTTTCCTAGTCAGAGTCAGC	ACCAAACCCACAGTCTTACAG
GAPDH	TCTCTGGCAAAGTCCAAGTG	TCACAAGTTTCCCGTTCTCAG

### Histopathology

For fixation, segments of the ileum were stippled on a cartoon piece to avoid bending, and immersed in Davidson's solution (100 mL glacial acetic acid, 300 mL 95% ethyl alcohol, 200 mL 10% neutral buffered formalin, and 300 mL distilled water), for at least 24 h. All the samples were transversally trimmed, dehydrated, infiltrated and embedded in paraffin, following common histological routine. Blocks were cut in 5 μm sections. All slides were stained with Hematoxylin and Eosin plus Alcian Blue in the same slide for goblet cells staining.

### ISI Methodology

The ISI methodology (5) was applied on the ileum samples from the two experiments, and the evaluated parameters are listed in Table 2. In this methodology, an impact factor (IF) is defined for each microscopic alteration according to the reduction of organ functional capacity, based on previous knowledge from the literature and background research. The IF ranges from 1 to 3, where IF = 3 is given to the most impactful alterations for the organ function (e.g., necrosis has the highest IF because the functional capacity of affected cells is totally lost). The extent of each lesion (intensity) or observed frequency compared to non-affected organs is evaluated though a score (S) that ranges from 0 to 3: score 0—absence of lesion or frequency; score 1—alteration up to 25% of the area or observed frequency; score 2—alteration ranges from 25 to 50% of the area or observed frequency and score 3—alteration extent more than 50% of the area or observed frequency.

**Table 2:** ISI histological alterations evaluated in ileum.

**Organ**	**Alteration**	**Impact factor (IF)**	Maximum^*^ score
Intestine	Lamina propria thickness (LPT)	2	45
	Epithelial thickness (ET)	1	
	Enterocytes proliferation (EP)	1	
	Inflammatory cell infiltration in the epithelium (EI)	1	
	Inflammatory cell infiltration in the lamina propria (LPI)	3	
	Goblet cells proliferation (GCP)	2	
	Congestion (CO)	2	
	Presence of *Eimeria* (PE)	3	

* Maximum score represents the sum of all alterations according to the formula ISI = Σ (IF*S), where IF = impact factor (previous fixed) and S = score (observed), considering the maximum observed S. For example, if a score S = 3 (maximum score) was observed for lamina propria thickness in a villus, this number must be multiplied by the IF of lamina propria thickness, which is 2. At the end, the ISI for this parameter in the villi will be ISI = (2*3) = 6. The average of 20 villi in the ileum for each bird will be the final ISI value for each bird (4).

To reach the ISI total score, which is the final value obtained through the methodology, the IF of each alteration is multiplied by the respective given score and the results of all alterations are summed, according to the formula ISI = *Σ* (IF × S). Twenty intestinal villi per bird were evaluated proportionally to the morphological distribution (merged and normal), in 10X objective (also used 40X objective to confirm alterations) of an optical microscope (Nikon Eclipse E200, São Paulo-SP- Brazil).

### Immunohistochemistry Analysis

The samples of liver and ileum were previously fixated in a zinc rich solution for 24 h in refrigerator (7, 8). The composition of the solution included calcium acetate, zinc acetate, zinc chloride and buffer with tris(hydroxymethyl)aminomethane (Tris) base (9). Then, they were immerged in alcohol 95% and progressively dehydrated, infiltrated and embedded in paraffin to make the tissue blocks. After the microtome sectioning at 4 μm, the samples were placed in immunohistochemistry slides, dewaxed in xylene at 60°C for 20 min and rehydrated in water and alcohol. The slides were horizontally placed in a humid incubation chamber, covered with 100 to 500 μL of primary specific antibodies (SouthernBiotech, USA) for avian macrophages, CD4+ and CD8+ T lymphocytes (added in different slides for each cell type) and incubated overnight at 5°C. Then, the slides were washed 3 times with phosphate buffered saline (PBS), covered with 100–500 μL of antibody conjugated with horseradish peroxidase (HRP-conjugated rabbit anti-mouse Ig, Dako North America, Carpinteria, CA, USA) and incubated for 30 min. The peroxidase reaction was developed using a chromogen for 30 s. The slides were counterstained with hematoxylin, washed in water, dehydrated, and mounted. The labeled cells were counted in an optical microscope (400X magnification objective). Five fields per bird were measured, totalizing 30 fields per treatment of intestine.

### Statistical Analyses

The data were processed by the software Statistix 9.0® (Analytical Software) submitted to Shapiro–Wilk normality test, followed by one-way analysis of variance (ANOVA) and Tukey *post-hoc* tests (*P* < 0.05). For the expression of cytokines, differences with *P* < 0.10 are also presented. For correlation analysis, Pearson's correlation coefficient (r) was used and the software provided the *P* values.

## Results

Gross lesions in the ileum mucosa of both treatments were occasional and comprised increased amount of mucous, mild hyperemia, and some edema in the mucosa. Lesions that characterize the Macroscopic Necrotic Enteritis were not observed. Microscopic morphological changes in ileum of both groups included congestion (CO) associated with a chronic mild enteritis characterized by inflammatory cells infiltrating the lamina propria (LPI), increasing its thickness (LPT), and inflammatory cells in the epithelium (EI). The proliferative changes were expressed by proliferation of immature enterocytes (EP) and goblet cells proliferation (GCP), both increasing the thickness of the epithelium (ET). Different stages of *Eimeria* development (macrogametocytes, microgametocytes, zygotes and oocysts) on and within the mucosa were only seen in the CH group. All of this tissue alterations have been translated in number for ISI methodology to allow a quantitative analysis for statistical comparison.

**Figure 1:**
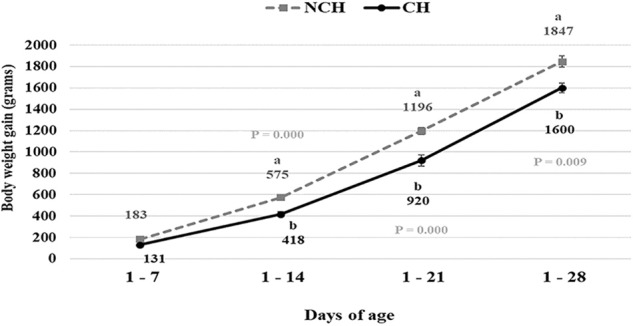
Body weight gain in non-challenged (NCH) and challenged (CH) groups. Bars represent the standard error of the mean. ^a,b^Different letters indicate significant difference at *P* < 0.05 at Tukey Test.

The CH group presented lower BWG at 1–14, 1–21, and 1–28 days (*P* < 0.01) (Figure 1) and worst FCR at 1–7 and 1–21 days of age (*P* < 0.05) (data not shown). No significant difference in FI was observed between groups at any age (data not shown).

**Figure 2:**
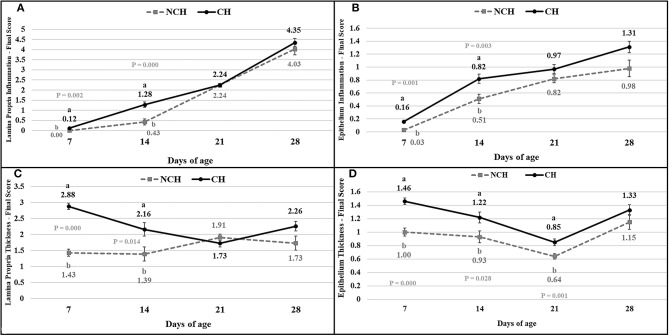
Lamina propria inflammation **(A)** epithelium inflammation **(B)**, lamina propria thickness **(C)**, epithelium thickness **(D)** in non-challenged (NCH) and challenged (CH) groups at 7, 14, 21, and 28 days of age. Bars represent the standard error of the mean. ^a,b^Different letters indicate significant difference at *P* < 0.05 at Tukey Test.

**Figure 3:**
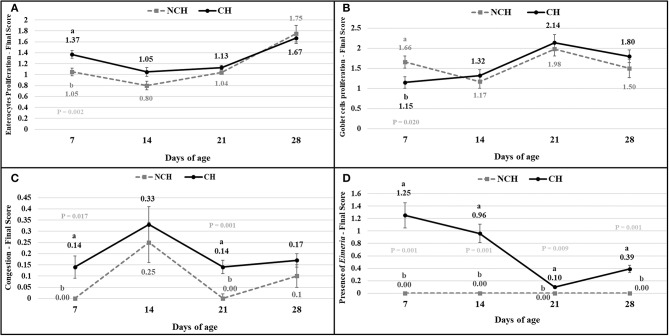
Enterocytes proliferation **(A)**, goblet cells proliferation **(B)**, congestion **(C)**, presence of *Eimeria* **(D)** in non-challenged (NCH) and challenged (CH) groups at 7, 14, 21, and 28 days of age. Bars represent the standard error of the mean. ^a,b^Different letters indicate significant difference at *P* < 0.05 at Tukey Test.

**Figure 4:**
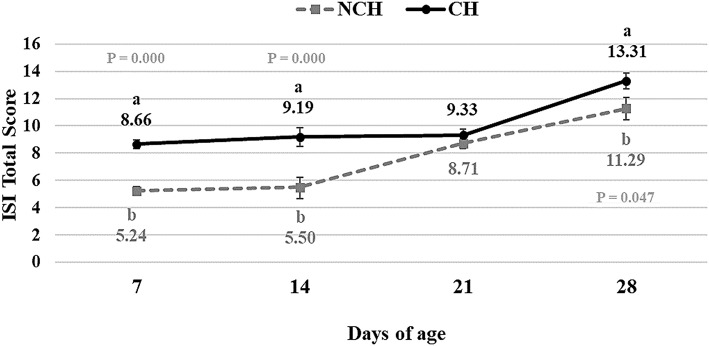
ISI Total Score in non-challenged (NCH) and challenged (CH) groups at 7, 14, 21, and 28 days of age. Bars represent the standard error of the mean. ^a,b^Different letters indicate significant difference at *P* < 0.05 at Tukey Test.

**Figure 5:**
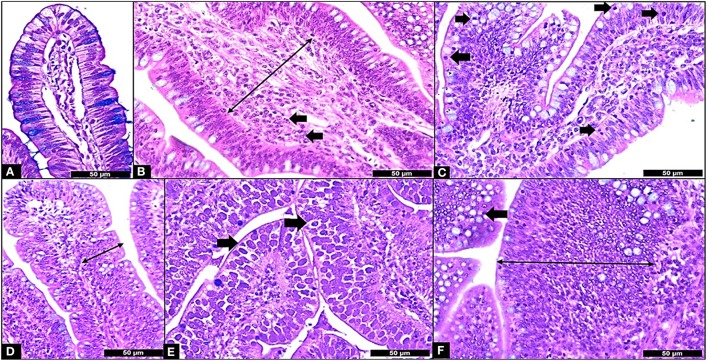
Microscopic Enteritis components and ISI parameters. **(A)** Normal villus; **(B)** Lamina propria thickness grade 2 (long arrow) and lamina propria inflammation (in arrows); **(C)** Epithelium inflammation (in arrows); **(D)** Epithelial thickness grade 2 (long arrow); **(E)** Presence of *Eimeria* in the mucosa (in arrows); **(F)** Goblet cells proliferation grade 2 (arrow) and enterocyte proliferation grade 3 (long arrow). All samples stained with Hematoxylin and Eosin plus Alcian Blue and 400X.

The results of the ileum histology by the ISI methodology are presented in chronological representation contrasting lamina propria inflammation (Figure 2A), epithelium inflammation (Figure 2B), lamina propria thickness (Figure 2C), epithelium thickness (Figure 2D), enterocytes proliferation (Figure 3A), goblet cells proliferation (Figure 3B), congestion (Figure 3C), presence of *Eimeria* spp. (Figure 3D) and the ISI total score (Figure 4) between groups. Some differences in the ileum histology are illustrated as photomicrographs in Figure 5.

From 7 to 28 days of age, it was possible to observe the development of inflammatory alterations in the ileum of both non-challenged and challenged animals. The chronological evaluation showed a similar tendency in the evolution of the ISI parameters in both groups, except for the presence of *Eimeria* spp. in the mucosa. However, the mixed infection by *Eimeria* spp. and CP intensified the expression of such inflammatory parameters in the challenged birds. This was verified through the higher ISI total score in the CH group at 7, 14, and 28 days of age in comparison to the NCH group (*P* < 0.05; Figure 4). At certain ages, the ileum of challenged broilers presented higher scores (*P* < 0.05) of lamina propria and epithelium inflammation, enterocytes proliferation, increased lamina propria thickness, increased epithelium thickness, and congestion and presence of *Eimeria* spp., but on the other hand, these birds presented lower scores of goblet cells proliferation (*P* < 0.05) (Figures 2, 3). The ISI histologic data was statistically correlated to the zootechnical performance (*P* < 0.05) as verifies through the Pearson's correlation. The ISI total score at 7 d was negatively correlated to BWG at 7 (*r* = −0.65), 14 (*r* = −0.80), 21 (*r* = −0.79), and 28 d (*r* = −0.79). In addition, the ISI total score at 14 d presented a negative correlation with BWG at 14 and 21 d (*r* = −0.61).

**Figure 6:**
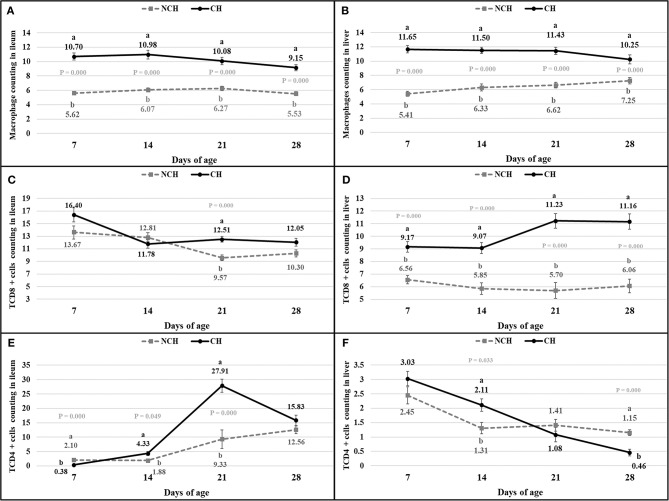
Count of macrophages **(A,B)**, CD8+ T cells **(C,D)**, and CD4+ T cells **(E,F)** in ileum **(A,C,E)** and liver **(B,D,F)** in non-challenged (NCH) and challenged (CH) groups at 7, 14, 21, and 28 days of age. Bars represent the standard error of the mean. ^a,b^Different letters indicate significant difference at *P* < 0.05 at Kruskal-Wallis Test.

The histologic alterations were accompanied by changes in the immune cell population of the ileal mucosa and liver. The challenge induced a higher count of macrophages in the ileum and liver of CH broilers in all ages when compared to animals from the NCH group (*P* < 0.001) (Figures 6A,B). The number of CD8+ T cells were also affected by the infection, once the CH group presented a higher count of CD8+ T cells in the ileum at 21 days and in the liver in all ages (*P* < 0.001) (Figures 6C,D). Challenged birds displayed an initial lower number of CD4+ T cells in ileum at 7 days (*P* < 0.001). However, an increase in the count of these leucocytes was verified in the following weeks. The ileal number of CD4+ T cells was higher in the CH group at 14 and 21 days (*P* < 0.05), with a peak in the latter (Figure 6E). An opposite situation was verified in the liver, where the hepatic count of CD4+ T cells in the CH birds was higher at 14 days (*P* < 0.05), but decreased at 28 days (*P* < 0.001), in comparison to the NCH group (Figure 6F).

**Figure 7:**
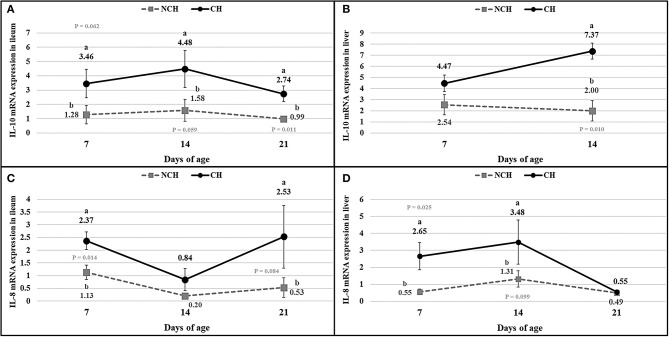
mRNA expression of interleukin-10 **(A,B)** and interleukin-8 **(C,D)** in ileum **(A,C)**, and liver **(B,D)** in non-challenged (NCH) and challenged (CH) groups at 7, 14, and 21 days of age (no data for IL-10 in liver at 21 days). Bars represent the standard error of the mean. ^a,b^Different letters indicate significant difference at *P* < 0.1 at Tukey Test.

The results of cytokines mRNA quantification (Figure 7) showed a higher expression of IL-8 mRNA in ileum and liver at 7 days (*P* < 0.05), in liver at 14 days (*P* < 0.10), and in ileum at 21 days (*P* < 0.10) in the CH group in comparison to the NCH birds. The IL-10 mRNA expression was higher in the ileum of challenged birds at 7, 14 (*P* < 0.10) and 21 days (*P* < 0.05) when compared to the non-challenged animals. In the liver, IL-10 mRNA was highly expressed in the CH group at 14 days (*P* < 0.05) in comparison to the NCH birds, but no level of expression was detected in either groups at 21 days.

## Discussion

In animal production, the enteritis here described through ISI parameters, is associated with a decrease in body weight gain, feed intake, feed efficiency and survivability (10, 11), however we observed a same trend in histological alteration in the ileum of non-challenged broilers. In human pathology, the idea of a microscopic inflammation with the absence of gross lesions has been accepted and described in patients with Celiac Disease (CD). This enteric condition has brought the concept of Microscopic Enteritis (ME), which is described by the Marsh classification in scores that range from 0 to 3 (12). The model proposed by Dr. Michael Marsh in 1992 lead to the first histological description of the human ME in chronic enteropathies and characterized the components of this condition as mucosal abnormalities without prominent inflammation (no gross lesions), villous effacement, erosions, or ulcerations on conventional light microscopy (2). In avian medicine, the microscopic evaluation of the gut has been made through the villi morphometry, where the height and width of villi and the depth and width of the crypts are measured. However, such analysis might not encompass the inflammatory events that impair the intestine, such as the infiltration of the gut mucosa by immune cells and the proliferative response of the mucosa, expressed as enterocytes and goblet cells proliferation and increase of villus layers or even the presence of parasites as different stages of *Eimeria* development (4, 5).

In the present study, we described a microscopic proliferative enteritis in broilers infected or not with *Eimeria* spp. and *Clostridium perfringens*. Although this mixed infection provokes an increased expression of the ISI histologic parameters, which is correlated to losses in zootechnical performance, the chronological evaluation of our data demonstrated a similar trend in the evolution of the inflammatory parameters in both challenged and non-challenged broilers, except for presence of *Eimeria* spp. in the mucosa. This finding suggests that broilers develop a microscopic basal enteritis (BE) during their life and more studies should be done to evaluate its effect on animal growth and health.

The enteritis promoted by the challenge is linked to expression of cytokines and changes in the immune cell population in the ileum mucosa and liver. The IL-10 is produced by many activated cells, including T lymphocytes, macrophages and keratinocytes. It was firstly described as a cytokine produced by T helper cells of subset 2 (Th2) and responsible for downregulating the expression of pro-inflammatory cytokines by Th1 cells, thus inhibiting the cell-mediated immunity (13). In fact, the IL-10 is important for regulating the inflammatory immune response and avoiding deleterious effects as tissue damage by the own leucocytes (14). Enterocolitis was a reality in mutant mice unable to express the IL-10 and this chronic inflammation was linked to an uncontrolled immune response to a variety of antigens present in the enteric lumen (13). Although the IL-10 has been proved to be essential in regulating the immune overreactions, pathogens can take advantage of this immunosuppressive property to evade the host immunity. The *Toxoplasma gondii*, for example, is capable of inhibiting the secretion of tumoral necrosis factor (TNF) while promoting the IL-10 expression (14). In chickens, the IL-10 was associated with susceptibility to *Eimeria maxima*, being highly expressed in the small-intestine of genetic lines more susceptible to protozoa, in comparison to those more resistant (15). In our study, the infected animals presented a higher expression of IL-10 mRNA in the ileum mucosa in all ages and in the liver at 14 days in comparison to the unchallenged birds (considering all differences with *P* < 0.10). The increased expression of this interleukin in the infected birds points its function on avoiding the excessive progression of the inflammatory response provoked by the challenge and the resulting dysbiosis in the gut. Collier et al. (16) verified that the co-infection by *E. acervulina, E. maxima* and CP elevates the expression of IL-10 in comparison to animals infected with protozoans only. In the present study, the inoculum of CP at 10, 11, and 12 days of age seemed to slightly increase the expression of IL-10 from 7 to 14 days. No expression of IL-10 mRNA was verified at 21 days, something probably linked to problems on the extraction of the genetic material.

Another cytokine assessed in our study was the interleukin-8. The IL-8 is an important neutrophil chemoattractant in mammals (17), also having a chemotactic effect in T cells (18, 19). In mice challenged with the *Clostridium difficile* toxin-α, the disturbed expression of IL-8 resulted in reduced scores of congestion and neutrophil infiltration, pointing its importance on the promotion of components of the acute immune response (20). A raised expression of IL-8 mRNA (considering all differences with *P* < 0.10) occurred in the challenged birds at 7 and 14 days in the liver and at 7 and 21 days in the ileum, jointly to the occurrence of increased scores of congestion. These events are possibly attributed to the acute immune responses induced by the challenges in the first and second weeks of age and the resulting modifications in the enteric microbiota.

While CD4+ T cells are responsible for regulating the immune response, CD8+ phenotype acts as a killer of infected cells (21). At 7 days, the challenged birds displayed a lower number of CD4+ T cells in the ileum mucosa in comparison to the unchallenged animals. However, an inverse result was verified on the two following weeks, with a growing higher quantity of these CD4+ T cells in the ileum of challenged birds. Chickens infected with *E. acervulina* or *E. tenella* and treated with anti-CD8 monoclonal antibodies (mab) presented augmented excretion of oocysts at 19–23 days before the primary infection (22). Similar results were verified in murine infected by *Eimeria pragensis*, where the treatment with anti-CD8 mab only provoked significant increase of oocyst output in the secondary infection, but not in the first challenge (23). Both groups of this study presented the highest numbers of CD8+ T lymphocytes in the ileum mucosa at 7 days of age, when the infected birds displayed a higher but not significantly different amount of these cells. While a decrease in this count occurred in unchallenged birds until 21 days, infected animals presented a significant infiltration of the mucosa by TCD8+ cells at this age. In addition, the lowest number of *Eimeria* sp. stages was verified in the ileum mucosa by the ISI evaluation at 21 days, when compared to the previous period evaluated at CH birds. This result might demonstrate the importance of TCD8+ lymphocytes in the resistance to later reinfection by ingestion of oocysts in the litter, as already demonstrated in the literature (22, 23). In all ages, the ileum and liver of infected animals are significantly infiltrated by macrophages. These cells are responsible for destructing pathogens through phagocytosis and for presenting antigens for T and B lymphocytes (24). Macrophages produce important pro-inflammatory molecules, including interleukin-1β (IL-1β), interferon-gamma (IFN-γ), tumoral necrosis factor-alpha (TNF-α), all up-regulated in eimerian infection (25, 26). Even in that case of an enteric disease, the liver presents itself as an immune organ, with its own dynamic of immune cells and of cytokine expression. In birds, the liver is known by its role as ectopic lymphoid tissue, mainly in the beginning of life (27).

According to the ISI, the first week of challenged was characterized by congestion and inflammation of the epithelium and lamina propria (LP) at 7 days in CH birds. At this early age, the villi of infected birds already display a proliferative response to the assault, with a proliferation of enterocytes and enlargement of the epithelium and LP. The number of goblet cells in the infected birds is lower in comparison to unchallenged ones at 7 days. These cells are responsible for producing mucus, which represent the first barrier of protection for the enteric surface, presenting a series of antimicrobial peptides (28). In mice infected with *Eimeria vermiformis*, the count of goblet cells reached the lowest number at 8 days post-infection (dpi) in the ileum, with a great recovery observed at 14 dpi (29). Similarly, murine infected with *E. papillate* had decreased number of goblet cells at 5 dpi in the jejunum, something linked to a lower expression of MUC-2 gene (30). The reduction of these cells might result from damages to the epithelium, from the assault against the multi-potential stem cells present in the crypts that originates the goblets (31). Once the challenge provoked a decrease in the goblet cell proliferation in the first week of age, the evolution of this parameter from 7 to 14 days in the challenged birds represents a recover in the population of these cells in the mucosa.

Both groups presented a continuous increase in the inflammation of epithelium and lamina propria from 7 to 28 days, pointing the presence of a microscopic basal enteritis as a form of response and defense of the mucosa. Other parameters as the enterocyte proliferation, epithelium thickness, congestion, and goblet cells proliferation (starting at 14 days), also present remarkable trend of progression in both groups. At 21 days, no difference is observed between groups for the inflammation of both epithelium and lamina propria, but infected animals still presented a higher epithelial thickness and congestion. This enlargement of the epithelium seems to be a late result of pathologic inflammation that occurred until 14 days, while the increased congestion might point the acute responses to the challenges in the first and second week, as discussed above.

Considering the environmental controlled condition at the present study (with no other external challenge) and the limited replication of vaccinal *Eimeria*, we can suggested that after the acute cycle of *Eimeria* and the development of a acquired immune response, both groups follow the same trend of basal enteritis that could be a regular condition in the broilers. Although the infection indeed decreases the zootechnical performance of the broilers, the basal enteritis observed in the non-challenged broilers rise the question of how this inflammation is affecting the zootechnical potential of the broilers at our currently poultry production.

More than describing an infectious pathologic process, the ISI methodology demonstrates that the inflammatory parameters of the ME evolve even in the absent of a specific pathogen, and this must be related to diet and components of the environment such as the litter. There is no clear mechanism to explain the BE and the hyperplasia of the epithelial cells in the NCH group. Some studies have attempted to classify the enteric inflammations as physiological (32), pathogenic with tissue destruction (33), metabolic (34), and sterile (35). Microscopically, the tissue destruction of the lamina propria by inflammation and its invasion by the proliferated immature enterocytes causing detachment of villi would certainly stimulate the chronicity of the process, suggesting pathological inflammation with regeneration as a mechanism of the ME. In addition, a possible consequence of the microscopic basal enteritis would be the permanent induction of the host defense (minor enteritis), leading to a breach of the intestinal epithelium and allowing *Salmonella* spp., by example, to interact with macrophages and heterophils (36).

More studies should be carried out, especially in field, to determine the value of the BE on animal zootechnical performance, health and well-being. Our data lead us to think about which changes in animal handling and nutrition could be made to reduce the number of inflammatory events translated by the ISI evaluation. If increase in ISI scores are related to performance losses, reduction on ISI could improve the zootechnical parameter and animal well-being while reducing the impact of poultry production for the environmental through improvement of feed efficacy.

## Data Availability Statement

The datasets generated for this study are available on request to the corresponding author.

## Ethics Statement

The animal study was reviewed and approved by Animal Use Ethics Commission (Comissão de Ética no Uso de Animais—CEUA), Agricultural Sciences Sector of the Federal University of Paraná.

## Author Contributions

All authors conducted the daily management of the animals, necropsies, and sampling. In addition, all authors performed the ISI microscopic evaluations of the samples. BB and IS managed the interleukin measurements assisted by AC. AS headed the ISI microscopic evaluation and the writing of the manuscript. ES organized the statistical calculations and performed the final review of the manuscript.

### Conflict of Interest

The authors in the present study are the group that developed the I See Inside methodology.

## References

[1] Shahraki T Rostami K Shahraki M Bold J Danciu M Dulaimi DA. Microscopic enteritis: clinical features and correlations with symptoms, 119-122. Gastroenterol Hepatol Bed Bench. (2012) 5:146–54. 24834216 PMC4017477

[2] Rostami K Villanacci V. Microscopic enteritis: novel prospect in coeliac disease clinical and immuno-histogenesis. Evolution in diagnostic and treatment strategies. Dig Liver Dis. (2009) 41:245–52. 10.1016/j.dld.2008.06.008 18657490

[3] Rostami K. Microscopic enteritis: bucharest consensus. World J Gastroenterol. (2015) 21:2593. 10.3748/wjg.v21.i9.2593 25759526 PMC4351208

[4] Kraieski AL Hayashi RM Sanches A Almeida GC Santin E. Effect of aflatoxin experimental ingestion and Eimeira vaccine challenges on intestinal histopathology and immune cellular dynamic of broilers:applying an Intestinal Health Index. Poult Sci. (2016) 96:1078–87. 10.3382/ps/pew397 27794052

[5] Belote BL Tujimoto-Silva A Hümmelgen PH Sanches AWD Wammes JCS Hayashi RM. Histological parameters to evaluate intestinal health on broilers challenged with Eimeria and *Clostridium perfringens* with or without enramycin as growth promoter. Poult Sci. (2018) 97:2287–94. 10.3382/ps/pey064 29660058

[6] Rostagno HS Albino LFT Donzele JL Gomes PC Oliveira RF Lopes DC Tabelas brasileiras Para aves e Suínos: Composição De Alimentos E Exigências Nutricionais. Viçosa: UFV University Press (2011). p. 3–252.

[7] Hicks DJ Johnson L Mitchell SM Gough J Cooley WA La Ragione RM. Evaluation of zinc salt based fixatives for preserving antigenic determinants for immunohistochemical demonstration of murine immune system cell markers. Biotech Histochem. (2006) 81:23–30. 10.1080/10520290600725375 16760124

[8] Mori H Soonsawad P Schuetter L Chen Q Hubbard NE Cardiff RD. Introduction of zinc-salt fixation for effective detection of immune cell related markers by immunohistochemistry. Toxicol Pathol. (2015) 43:883–89. 10.1177/0192623315587593 26157038 PMC4539256

[9] Beckstead JH. A simple technique for preservation of fixation-sensitive antigens in paraffin-embedded tissues. J Histochem Cytochem. (1994) 42:1127–34. 10.1177/42.8.8027531 8027531

[10] Iseri VJ Klasing KC. Dynamics of the systemic components of the chicken. (Gallus gallus domesticus) immune system following activation by *Escherichia coli*; implications for the costs of immunity. Dev Comp Immunol. (2013) 40:248–57. 10.1016/j.dci.2013.02.005 23500513

[11] Kogut MH Genovese KJ Swaggerty CL He H Broom L. Inflammatory phenotypes in the intestine of poultry:not all inflammation is created equal. Poult Sci. (2018) 97:2339–46. 10.3382/ps/pey087 29618086

[12] Marsh MN. Gluten, major histocompatibility complex, and the small intestine. Gastroenterology. (1992) 102:330–54. 10.1016/0016-5085(92)91819-P 1727768

[13] Kühn R Löhler J Rennick D Rajewsky K Müller W. Interleukin-10-deficient mice develop chronic enterocolitis. Cell. (1993) 75:263–74. 10.1016/0092-8674(93)80068-P 8402911

[14] Cyktor JC Turner J. Interleukin-10 and immunity against prokaryotic and eukaryotic intracellular pathogens. Infect Immun. (2011) 79:2964–73. 10.1128/IAI.00047-11 21576331 PMC3147550

[15] Rothwell L Young JR Zoorob R Whittaker CA Hesketh P Archer A. Cloning and characterization of chicken IL-10 and its role in the immune response to eimeria maxima. J Immunol. (2004) 173:2675–82. 10.4049/jimmunol.173.4.2675 15294985

[16] Collier CT Hofacre CL Payne AM Anderson DB Kaiser P Mackie RI. Coccidia- induced mucogenesis promotes the onset of necrotic enteritis by supporting *Clostridium perfringens* growth. Vet Immunol Immunopathol. (2008) 122:104–15. 10.1016/j.vetimm.2007.10.014 18068809

[17] Harada A Sekido N Akahoshi T Wada T Mukaida N Matsushima K. Essential involvement of interleukin-8. (IL-8) in acute inflammation. J Leukoc Biol. (1994) 56:559–64. 10.1002/jlb.56.5.559 7964163

[18] Larsen C Anderson A Appella E Oppenheim J Matsushima K. The neutrophil-activating protein. (NAP-1) is also chemotactic for T lymphocytes. Science. (1989) 243:1464–6. 10.1126/science.2648569 2648569

[19] Taub DD Anver M Oppenheim JJ Longo DL Murphy WJ. T lymphocyte recruitment by interleukin-8. (IL-8). IL-8-induced degranulation of neutrophils releases potent chemoattractants for human T lymphocytes both *in vitro* and *in vivo*. J Clin Invest. (1996) 97:1931–41. 10.1172/JCI118625 8621778 PMC507263

[20] Lee JY Park HR Oh Y-K Kim Y-J Youn J Han J-S. Effects of transcription factor activator protein-1 on interleukin-8 expression and enteritis in response to *Clostridium difficile* toxin A. J Mol Med. (2007) 85:1393–404. 10.1007/s00109-007-0237-7 17639289

[21] Kaiser P. Advances in avian immunology—prospects for disease control:a review. Avian Pathol. (2010) 39:309–24. 10.1080/03079457.2010.508777 20954007

[22] Trout JM Lillehoj HS. T lymphocyte roles during *Eimeria acervulina* and *Eimeria tenella* infections. Vet Immunol Immunopathol. (1996) 53:163–72. 10.1016/0165-2427(95)05544-4 8941977

[23] Rose ME Hesketh P Wakelin D. Immune control of murine coccidiosis: CD4+ and CD8+ T lymphocytes contribute differentially in resistance to primary and secondary infections. Parasitology. (1992) 105:349–54. 10.1017/S0031182000074515 1361049

[24] Dalloul RA Lillehoj HS. Poultry coccidiosis: recent advancements in control measures and vaccine development. Exp Rev of Vaccines. (2006) 5:143–63. 10.1586/14760584.5.1.143 16451116

[25] Byrnes S Eaton R Kogut M. *In vitro* interleukin-1 and tumor necrosis factor-alpha production by macrophages from chickens infected with either *Eimeria maxima* or *Eimeria tenella*. Int J Parasitol. (1993) 23:639–45. 10.1016/0020-7519(93)90170-4 8225766

[26] Laurent F Mancassola R Lacroix S Menezes R Naciri M. Analysis of chicken mucosal immune response to *Eimeria tenella* and *Eimeria maxima* infection by quantitative reverse transcription-PCR. Infect Immun. (2001) 69:2527–34. 10.1128/IAI.69.4.2527-2534.2001 11254616 PMC98188

[27] Oláh I Nagy N Vervelde L Structure of the avian lymphoid system. In: Davison F Kaspers B Schat KA, editors. Avian Immunology, 1st Edn. Elsevier Inc (2008). p. 13–50. 10.1016/B978-0-12-370634-8.X5001-X

[28] Hansson GC. Role of mucus layers in gut infection and inflammation. Curr Opin in Microbiol. (2012) 15:57–62. 10.1016/j.mib.2011.11.002 22177113 PMC3716454

[29] Linh BK Hayashi T Horii Y. *Eimeria vermiformis* infection reduces goblet cells by multiplication in the crypt cells of the small intestine of C57BL/6 mice. Parasitol Res. (2008) 104:789–94. 10.1007/s00436-008-1256-1 19005680

[30] Dkhil MA Delic D Al-Quraishy S. Goblet cells and mucin related gene expression in mice infected with *Eimeria papillata*. Sci World J. (2013) 2013:1–6. 10.1155/2013/439865 24367242 PMC3866723

[31] Shaoul R Hong D Okada Y Cutz E Marcon MA. Lineage development in a patient without goblet, paneth, and enteroendocrine cells: a clue for intestinal epithelial differentiation. Pediatr Res. (2005) 58:492–8. 10.1203/01.PDR.0000179408.74781.C9 16148062

[32] Abraham C Medzhitov R. Interaction between the host innat immune system and microbes in inflammatory bowel disease. Gastroenterology. (2011) 140:1729–37. 10.1053/j.gastro.2011.02.012 21530739 PMC4007055

[33] Medzhitov R. Origin and physiological roles of inflammation. Nature. (2008) 454:428–35. 10.1038/nature07201 18650913

[34] Hotamisligil GS. Inflammation and metabolic disorders. Nature. (2006) 444:860–7. 10.1038/nature05485 17167474

[35] Rubartelli A Lotze MT Latz E Manfredi A. Mechanisms of sterile inflammation. Front Immunol. (2013) 4:article 398. 10.3389/fimmu.2013.00398 24319446 PMC3837241

[36] Wigley P. *Salmonella enterica* in the chicken: how it has helped our understanding of immunology in a non-biomedical model species. Front Immunol. (2014) 5:482. 10.3389/fimmu.2014.00482 25346731 PMC4193332

